# Supplementary material for: Complexome profiling of the Chlamydomonas psb28 mutant reveals TEF5 as an early PSII assembly factor
Source: Plant Cell. 2025 Mar 17;37(6):koaf055. doi: 10.1093/plcell/koaf055 (PMC12164590; doi:10.1093/plcell/koaf055)
Supplement: koaf055_Supplementary_Data [file koaf055_supplementary_data.zip › psb28_interactive.zip!/plots/intensity/qualityPlots/peptides/_Cre12.g547500.t1.2.html]

### Cre12.g547500.t1.2

Trivial name:   
SeqCoverage [median]: 31.60  
Mass: 11.79 kDa  
  
 

| Sequence | MissedCleavage | Mass [Da] | wtIntensity | muIntensity | Score |
| --- | --- | --- | --- | --- | --- |
| ASGDRGPGAFGNDGNSDVVFVAK | 1 | 2236.05 | 0.00 | 1145.37 | 44.26 |
| GPGAFGNDGNSDVVFVAK | 0 | 1749.83 | 0.00 | 1423.72 | 58.83 |
|  |  |  | 0.0 | 2569.1 |  |
